# Time-Dependent Polystyrene Nanoplastic Toxicity in *Cherax quadricarinatus*: Oxidative Stress, Gut Dysbiosis, and Hepatopancreatic Bioaccumulation

**DOI:** 10.3390/ani16131977

**Published:** 2026-06-26

**Authors:** Shun Cheng, Hai-Heng Wang, Mei-Li Chi, Wen-Ping Jiang, Shi-Li Liu, Wen-Wu Zou, Zhi-Long Chen, Fei Li

**Affiliations:** 1 Key Laboratory of Healthy Freshwater Aquaculture, Zhejiang Institute of Freshwater Fisheries (Zhejiang Freshwater Fishery Environmental Monitoring Station), Ministry of Agriculture and Rural Affairs, Huzhou 313001, China; sschengshun@sina.com (S.C.); chimeili83404109@126.com (M.-L.C.); jordanping23@icloud.com (W.-P.J.); liushili1212@126.com (S.-L.L.); 2 Wencheng County Modern Agriculture and Kangyang Industry Research Institute, Wencheng, Wenzhou 325300, China; dream_heng@126.com (H.-H.W.); wcnky2018@163.com (W.-W.Z.); zhilongchen@sina.com (Z.-L.C.)

**Keywords:** redclaw crayfish, polystyrene nanoplastics, enzyme activity, intestinal microbes, hepatopancreatic enrichment

## Abstract

Polystyrene nanoplastic (PS-NP) contamination poses an emerging threat to aquaculture species, yet time-resolved assessments integrating host physiology, gut microbial ecology, and tissue bioaccumulation remain limited. Here, we evaluated the temporal effects of 100 mg/L PS-NPs (100 nm) on *Cherax quadricarinatus* (Von Martens, 1868) over a 3-week exposure period. Crayfish were assigned to a control group (Group A) and three treatment groups exposed for 1 (Group B), 2 (Group C), or 3 (Group D) weeks. No mortality occurred. Hepatopancreatic antioxidant enzyme activities (superoxide dismutase and glutathione peroxidase) displayed a hormetic response (upregulation at weeks 1–2 followed by depletion at week 3), indicating oxidative stress overload. Alkaline phosphatase activity declined progressively, reflecting cumulative immunosuppression. Histological examination revealed time-dependent structural damage in the hepatopancreas: hepatic tubule enlargement, increased vacuolation, B cell hypertrophy, and cellular disorganization/lysis after three weeks. 16S rRNA sequencing revealed that PS-NPs induced time-dependent gut dysbiosis, characterized by depletion of beneficial taxa and enrichment of opportunistic pathogens. Alpha-diversity metrics (ACE, Chao1, Shannon) were significantly reduced in Group D compared to controls, confirming loss of microbial evenness and richness. Pyrolysis gas chromatography–mass spectrometry quantification demonstrated marked PS-NP bioaccumulation in the hepatopancreas, with concentrations rising from 6.94 μg/g in controls to 65.38 μg/g in Group D, a 9.4-fold increase. Collectively, prolonged PS-NP exposure is associated with oxidative stress, immune dysfunction, progressive gut dysbiosis, and substantial hepatopancreatic nanoplastic burden in *C. quadricarinatus.* These findings carry implications for ecological risk assessment and highlight the need for further investigation into food safety risks associated with human consumption of crayfish from PS-NP-contaminated environments.

## Simple Summary

Microplastic pollution is a growing environmental problem that can harm aquatic animals. In this study, we investigated how polystyrene microplastics (PS-NPs) affect redclaw crayfish (*Cherax quadricarinatus*), a commercially important freshwater species, over three weeks of exposure. PS-NPs caused time-dependent damage to the crayfish. After three weeks, the crayfish showed signs of oxidative stress and weakened immune function in their hepatopancreas, a digestive and detox organ. Microscopic examination revealed progressively structural damaged, with cells swelling, forming abnormal vesicles, and eventually breaking down. PS-NPs also altered the gut bacterial communities: beneficial bacteria decreased while potentially harmful bacteria increased, reducing overall microbial diversity. In addition, the hepatopancreas accumulated large amounts of plastic particles, reaching nearly ten times the level found in unexposed crayfish. These findings show that long-term exposure to nanoplastics can seriously affect the health of crayfish. Because the hepatopancreas is often eaten by people, our results also raise caution about possible food safety risks when consuming crayfish from waters contaminated with nanoplastics.

## 1. Introduction

The pervasive contamination of aquatic ecosystems by polystyrene nanoplastics (PS-NPs) has emerged as a critical ecological issue, exerting deleterious effects on cultured aquatic organisms [1]. PS-NPs primarily originate from the physicochemical degradation of macroplastic debris, thereby contributing to the progressive deterioration of water quality [2]. Projections indicate that environmental microplastic loading may increase by 1.5- to 2.5- fold by 2040; moreover, even under a scenario of complete cessation of new plastic emissions, the continued fragmentation of legacy plastic polymers will sustain microplastic accumulation for the foreseeable future [3]. Consequently, elucidating the toxicological implications of PS-NP exposure and their bioaccumulation potential in aquatic biota is of paramount importance.

Polystyrene (PS) warrants particular attention among plastic polymers for two main reasons. First, PS is one of the most abundantly produced plastics globally (≈7% of total plastic production) and is widely used in disposable products, foam packaging, and insulation materials. Its brittle nature makes it highly susceptible to environmental fragmentation, contributing substantially to the micro- and nanoplastic burden in aquatic systems [4]. Indeed, alongside polyethylene and polypropylene, PS is consistently identified as a dominant polymer type in freshwater environments [5]. Second, PS particles, particularly in the nano-size range, have been shown to induce oxidative stress, immunotoxicity, and gut microbiota dysbiosis in aquatic organisms. As a result, PS-based particles have become the most frequently used model materials in nanoplastic toxicity research [5].

Monodisperse spherical PS-NPs (100 nm) were selected as model particles in this study. The majority of nanoplastic toxicity studies to date have employed such monodisperse polystyrene nanospheres, primarily due to their commercial availability, well-characterized physicochemical properties (size, shape, surface charge), and batch-to-batch consistency, which facilitate cross-study comparisons and mechanistic investigations [6]. While PS may not inherently possess higher toxicity than other polymers—some studies report, for instance, that polyvinyl chloride induces stronger intestinal toxicity in fish [7]—ts widespread use as a reference material allows the present findings to be contextualized within the broader literature. Nevertheless, exclusive reliance on a single polymer type and particle shape represents a limitation, as environmentally occurring MNPs exhibit diverse physicochemical characteristics.

*Cherax quadricarinatus* (Von Martens, 1868) (redclaw crayfish) has emerged as an economically significant freshwater decapod, with its aquaculture production expanding markedly across China in recent years [8,9,10]. As a benthic opportunistic feeder, this species exhibits heightened susceptibility to incidental ingestion of sediment-associated microplastics [9]. Given the potential ramifications of PS-NP contamination for both aquaculture sustainability and consumer safety, targeted investigations into the physiological and ecological responses of *C. quadricarinatus* to nanoplastic stress are urgently warranted.

Accumulating evidence indicates that crustaceans are particularly vulnerable to PS-NP toxicity. Existing studies have predominantly focused on histopathological lesions [11], oxidative stress [12], and behavioral impairments [13] across various decapod species. Nevertheless, comprehensive assessments integrating systemic toxicological endpoints with quantitative bioaccumulation data remain scarce. For instance, chronic exposure to 10 mg/L PS-NPs over four weeks induced apoptosis, autophagy, histological damage, and gut microbiota dysbiosis in *Litopenaeus vannamei* [14]. PS-NPs have also been implicated in hepatotoxicity via bioaccumulation and reactive oxygen species (ROS)-mediated oxidative injury in penaeid shrimp [12,15]. Similarly, exposure to 20 mg/L PS-NPs (75 nm) disrupted intestinal microbial homeostasis in *Procambarus clarkii*, potentially compromising host immune competence [16], while parallel studies documented pronounced hepatopancreatic histopathology in *L. vannamei* [15]. Regarding *C. quadricarinatus*, preliminary investigations by Zhu et al. demonstrated that 100 nm PS-NPs at concentrations up to 100 μg/L elicited histological aberrations and oxidative stress [17]. Despite these advances, a holistic evaluation of the pleiotropic effects of PS-NPs encompassing enzymatic dysfunction, microbial community restructuring, and tissue-specific bioaccumulation, remains conspicuously absent for this commercially important species.

The hepatopancreas, the largest metabolic organ in decapod crustaceans, plays an indispensable role in xenobiotic detoxification and nutrient metabolism [15]. Previous studies have documented differential accumulation of PS-NPs across tissues in *L. vannamei*, with the highest burdens consistently localized to the hepatopancreas. Such bioaccumulation has been causally linked to growth retardation, aberrant swimming behavior, and diminished locomotor performance [12]. Beyond its ecotoxicological relevance, microplastic contamination poses direct human health concerns: ingested PS-NPs can translocate across the gastrointestinal barrier and accumulate in vital organs (including the lungs, liver, and brain) with emerging evidence implicating chronic exposure in carcinogenic processes [18]. The hepatopancreas, frequently consumed as a delicacy in many cultures, thus represents a potential vector for dietary microplastic exposure. Accordingly, quantifying PS-NP enrichment in this tissue is essential for informing evidence-based food safety guidelines and mitigating public health risks.

In light of these knowledge gaps, the present study was designed to systematically evaluate the time-dependent effects of PS-NPs on *C. quadricarinatus* across multiple biological levels. By exposing crayfish to 100 mg/L PS-NPs for 1, 2, and 3 weeks, we aimed to characterize the temporal dynamics of antioxidant and immune enzyme activities, gut microbiota compositional shifts, and hepatopancreatic microplastic accumulation. This integrative approach not only addresses a critical gap in crustacean nanoplastic toxicology but also generates actionable data for aquaculture risk assessment and consumer protection.

## 2. Materials and Methods

### 2.1. Experimental C. quadricarinatus and PS-NP Exposure

The toxicity bioassay was conducted at the Zhejiang Institute of Freshwater Fisheries (Huzhou, China). Specimens of *C. quadricarinatus* were obtained from the institute’s breeding facility. Polystyrene nanoplastic (PS-NPs) with a nominal diameter of 100 nm and a density of 1.05 g/cm^3^ were purchased as an aqueous stock suspension (2.5% *w*/*v*, 10 mL) from Tianjin Beile Chromatography Technology Development Center (Tianjin, China).

A total of 72 intermolt crayfish (mean body weight: 67.99 ± 12.84 g; mean body length: 12.30 ± 0.82 cm) were used. Individuals were housed in breeding tanks (0.21 m^2^ base area) containing 14.74 L of dechlorinated tap water. Water temperature was maintained at 22.0 ± 1.0 °C under a 12 h light: 12 h dark photoperiod. Continuous aeration ensured dissolved oxygen levels remained between 6.5 and 7.5 mg/L throughout the acclimation and exposure periods.

After a 7-day acclimation, crayfish were randomly assigned to four experimental groups, each with three replicate tanks (*n* = 3 tanks per group, 6 crayfish per tank):

Group A (Control): No PS-NP exposure.

Group B (1 week): Exposed to 100 mg/L PS-NPs for 1 week.

Group C (2 weeks): Exposed to 100 mg/L PS-NPs for 2 weeks.

Group D (3 weeks): Exposed to 100 mg/L PS-NPs for 3 weeks.

To eliminate dietary interference, crayfish were fasted throughout the exposure period. To maintain the nominal PS-NP concentration, 100% water renewal was performed weekly, with immediate re-addition of PS-NPs to the target concentration.

At the termination of each exposure interval (weeks 1, 2, and 3), survival was recorded. At each time point, six crayfish from the control group (Group A) and six crayfish from the respective exposure group (Group B at week 1, Group C at week 2, Group D at week 3) were anesthetized with 100 mg/L MS-222 and euthanized by rapid decapitation following institutional ethical guidelines.

Hepatopancreas and intestinal tissues were immediately dissected on an ice-cooled platform. Samples intended for enzymatic and microbiota analyses were snap-frozen in liquid nitrogen and stored at −80 °C until processing. Additional hepatopancreas samples were fixed in Bonn’s solution for histopathological examination.

To minimize background nanoplastic contamination, all glassware and tanks were thoroughly rinsed with deionized water filtered through 0.22 μm membranes. Experimental water was pre-filtered through a 0.45 μm polycarbonate membrane to remove pre-existing particles. Procedural blanks (deionized water subjected to the same handling steps) were collected weekly and analyzed to monitor potential airborne or cross-contamination.

All experimental procedures were approved by the Ethics Committee of the Laboratory Animal Center, Zhejiang Institute of Freshwater Fisheries.

### 2.2. Analytical Procedures

#### 2.2.1. Tissue Processing

At each sampling time point (1, 2, and 3 weeks), six crayfish per group were euthanized. The hepatopancreas and the entire intestinal tract were dissected under sterile conditions.

For histopathological observation, hepatopancreas samples were fixed in Bonn’s solution for 24 h, then dehydrated through a graded ethanol series, cleared in xylene, and embedded in paraffin wax. Sections of 5 µm thickness were cut using a microtome, stained with hematoxylin and eosin (H&E), and mounted with neutral gum.

Separately, the intestine was opened longitudinally with sterile scissors, and the luminal contents were gently scraped using a sterile scalpel blade. All tissue and content samples (remaining hepatopancreas after sampling for histology, and intestinal contents) were immediately frozen in liquid nitrogen and stored at −80 °C pending further analysis.

#### 2.2.2. Quantification of Antioxidant and Immune-Related Enzyme Activities

Hepatopancreas samples were homogenized in ice-cold physiological saline (1:9, *w*/*v*) using a motor-driven tissue homogenizer. Homogenates were centrifuged at 1000× *g* for 10 min at 4 °C, and the resultant supernatants were aliquoted and maintained at 4 °C for immediate enzymatic assays. Activities of superoxide dismutase (SOD), glutathione peroxidase (GPx), acid phosphatase (ACP), and alkaline phosphatase (AKP) were quantified using commercial colorimetric kits (Nanjing Jiancheng Bioengineering Institute, Nanjing, China) strictly following the manufacturer’s protocols. All assays were performed in triplicate, and kits from a single production batch were used to minimize inter-assay variability.

#### 2.2.3. Gut Microbiota Profiling

Total genomic DNA was extracted from the intestinal content samples using the QIAamp DNA Stool Mini Kit (Qiagen, Hilden, Germany) according to the manufacturer’s instructions. The V3-V4 hypervariable region of the bacterial 16S rRNA gene was amplified using universal primers 338F (5′-ACTCCTACGGGAGGCAGCAG-3′) and 806R (5′-GGACTACHVGGGTWTCTAAT-3′). PCR products were purified, quantified, and pooled for library construction. Paired-end sequencing (2 × 300 bp) was performed on an Illumina MiSeq platform (Illumina, San Diego, CA, USA).

Raw sequencing reads were demultiplexed, quality-filtered using QIIME2, and merged. High-quality sequences were clustered into operational taxonomic units (OTUs) at 97% sequence similarity using UPARSE. Taxonomic assignment was performed against the Silva 138.1 database. Alpha diversity metrics (ACE, Chao1, Shannon, Simpson) were calculated based on rarefied OTU tables. Beta diversity was assessed via principal coordinate analysis (PCoA) based on weighted UniFrac distances.

#### 2.2.4. Quantification of Nanoplastic Accumulation in Hepatopancreas

The accumulation of 13 common polymer types, including polystyrene (PS), polyethylene (PE), polypropylene (PP), poly (methyl methacrylate) (PMMA), polyvinyl chloride (PVC), polyethylene terephthalate (PET), polycarbonate (PC), polyamide 6 (PA6), polyamide 66 (PA66), polylactic acid (PLA), poly (butylene adipate-co-terephthalate) (PBAT), polyurethane (PU), and polyoxymethylene (POM), was quantified using pyrolysis gas chromatography–mass spectrometry (Py-GC/MS). Hepatopancreas samples from Groups A and D were subjected to solvent extraction followed by concentration under a gentle nitrogen stream. Aliquots of the concentrated extract were transferred to deactivated pyrolysis cups and air-dried prior to analysis.

Analysis was performed on a GCMS-QP2020 system (Shimadzu, Kyoto, Japan) coupled with a PY-3030D multi-shot pyrolyzer (Frontier Laboratories, Koriyama, Fukushima, Japan). The pyrolysis temperature was set at 550 °C with a split ratio of 5:1. Helium was used as the carrier gas. Chromatographic separation was achieved on an Rtx-5MS capillary column (30 m × 0.25 mm i.d., 0.25 μm film thickness). The oven temperature program was as follows: hold at 40 °C for 2 min, ramp to 320 °C at 20 °C/min, and hold for 14 min (total run time: 30 min). The ion source temperature was maintained at 230 °C, and mass spectra were acquired in full-scan mode (*m*/*z* 29–600). Quantification was performed using external calibration curves constructed from authentic polymer standards.

To ensure analytical reliability, procedural blanks (solvent extracts without tissue) and instrument blanks (pure helium injections) were analyzed every 10 samples. Recovery rates were determined by spiking control hepatopancreas samples with known amounts (10, 50, and 100 μg/g) of reference PS-NPs; mean recoveries ranged from 78.2% to 93.5% (RSD < 12%). All reported concentrations were corrected against the average blank values, which were consistently below the limit of detection (0.1 μg/g) for all target polymers.

Quantification of microplastic accumulation was conducted on hepatopancreas samples from the control group (Group A) and the 3-week exposure group (Group D). Samples from Groups B and C were not analyzed due to limited sample material and analytical resource constraints.

### 2.3. Statistical Analysis

All data were expressed as mean ± standard deviation (SD). Prior to analysis, percentage data were arcsine-square root transformed where necessary to meet normality assumptions. Differences among treatment groups were evaluated using one-way analysis of variance (ANOVA) followed by Tukey’s honestly significant difference (HSD) post hoc test for multiple comparisons. Statistical analyses were conducted using SPSS software (version 17.0), with significance set at *p* < 0.05. Figures were generated using GraphPad Prism (version 8.0) and R (version 4.1.0) with packages “ggplot2”, “vegan”, and “pheatmap”. Microbiota sequencing data were processed using QIIME2 (version 2020.11) and UPARSE (version 11).

## 3. Results

### 3.1. Survival

No mortality was observed in any treatment group throughout the experimental period, resulting in a 100% survival rate across all groups.

### 3.2. Effects of Exposure Duration on Antioxidant and Immune-Related Indicators in the Hepatopancreas

The activities of antioxidant and immune-related enzymes in the hepatopancreas exhibited distinct time-dependent responses to PS-NP exposure.

**Figure 1:**
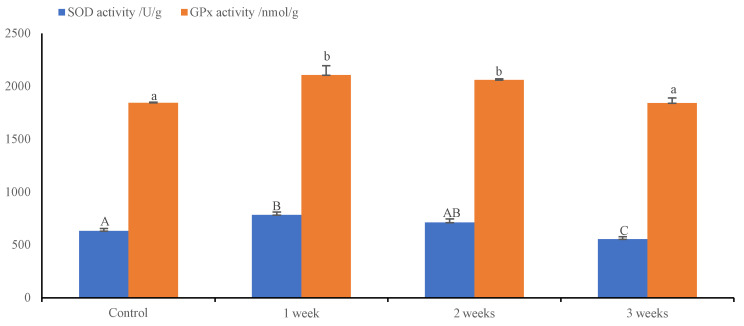
Effects of exposure duration on the activities of antioxidant enzymes in the hepatopancreas of *C. quadricarinatus* exposed to PS-NPs. Different lowercase and uppercase letters above bars indicate significant differences among groups (*p* < 0.05).

Antioxidant enzymes: The activities of both SOD and GPx showed an initial increase followed by a subsequent decrease with prolonged exposure (Figure 1). Specifically, SOD activity in Group B (1-week exposure) (781.04 ± 30.32 U/g) was significantly higher than in the control Group A (631.45 ± 22.67 U/g) (*p* < 0.05). However, after 3 weeks of exposure (Group D), SOD activity (552.74 ± 24.01 U/g) decreased significantly and was lower than in Group B and C (711.13 ± 34.28 U/g) (*p* < 0.05). A similar pattern was observed for GPx activity, where Groups B (2104.49 ± 89.00 nmol/g) exhibited significantly higher activities compared to both the control (Group A) (1843.15 ± 6.50 nmol/g) and the 3-week exposure group (Group D) (1839.86 ± 49.76 nmol/g) (*p* < 0.05). After 3 weeks of exposure (Group D), GPx activity decreased significantly and was lower than in all other groups (*p* < 0.05).

**Figure 2:**
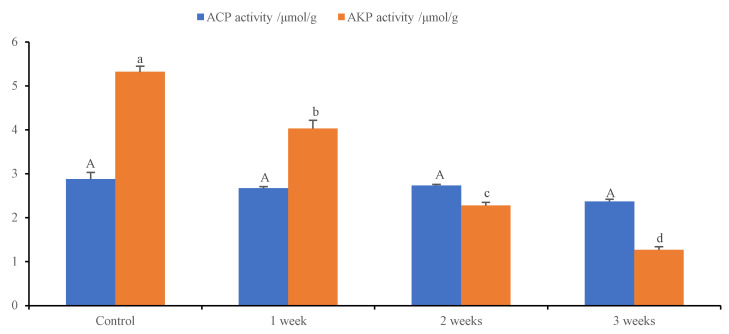
Effects of exposure duration on the activities of Immune-related enzymes in the hepatopancreas of *C. quadricarinatus* exposed to PS-NPs. Different lowercase and uppercase letters above bars indicate significant differences among groups (*p* < 0.05).

Immune-related enzymes: In contrast to the antioxidant enzymes, ACP activity remained stable and showed no significant differences among the four groups (*p* > 0.05) (Figure 2). Conversely, AKP activity displayed a consistent decline with increasing exposure time. AKP activity was significantly highest in the control Group A (5.32 ± 0.13 μmol/g) and significantly lowest in Group D (3-week exposure) (1.27 ± 0.07 μmol/g) (*p* < 0.05), indicating a pronounced inhibitory effect of prolonged PS-NP stress on this immune parameter.

### 3.3. Effects of PS-NP Exposure on Hepatopancreas Histology

**Figure 3:**
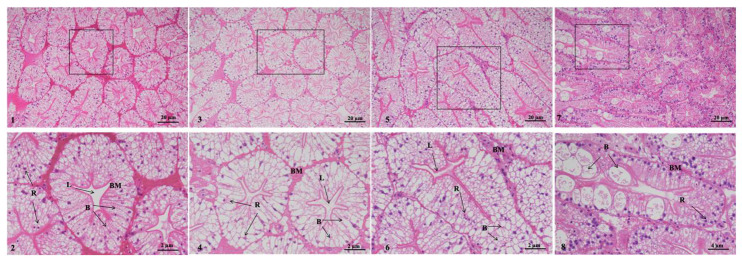
Effects on hepatopancreas microstructure of *C. quadricarinatus* under stress. B, B cell; R, R cell; BM, Basement membrane; L, lumen. The numbered images represent the treatment groups: **1** and **2** represent Group A; **3** and **4**, Group B; **5** and **6**, Group C; **7** and **8**, Group D.

Histopathological examination of the hepatopancreas revealed progressive structural damage with increasing PS-NP exposure duration (Figure 3).

In the control group (Group A), the hepatic tubules exhibited intact basement membranes with clearly defined boundaries and a star-shaped central lumen. Hepatopancreatic cells were normally structured, evenly distributed, and tightly packed. After one week of exposure (Group B), the hepatic tubules remained relatively normal in architecture. B cells (secretory cells) and R cells (absorptive/storage cells) were evenly distributed, although some B cells showed an increase in cell volume. Following two weeks of exposure (Group C), the hepatic tubules became significantly enlarged and appeared flattened, extending laterally. The number and size of intracellular vacuoles increased. The arrangement of hepatopancreatic cells became disorganized. B cells exhibited a marked increase in both cell volume and the number of intracellular transport vesicles, with some vesicles containing visible particulate matter. After three weeks of exposure (Group D), the hepatic tubules were further enlarged, flattened, and displayed blurred boundaries. B cells and their transport vesicles continued to increase in volume. The particulate material within transport vesicles became substantially more abundant. This vesicle enlargement caused morphological distortion of the hepatic tubules, leading to compression and deformation of the lumen. Cellular arrangement was severely disrupted, hepatopancreatic cells showed signs of lysis, and cell numbers were markedly reduced compared to other groups.

### 3.4. Effects of Exposure Duration on Gut Microbiota

#### 3.4.1. Microbial Diversity Indices

High-throughput sequencing of the 16S rRNA gene yielded 1,057,575 high-quality reads from 12 intestinal samples, which were subsequently clustered into 650 operational taxonomic units (OTUs) at 97% sequence similarity. Taxonomic assignment revealed the presence of 25 phyla, 54 classes, 124 orders, 207 families, 340 genera, and 391 species across all samples.

**Figure 4:**
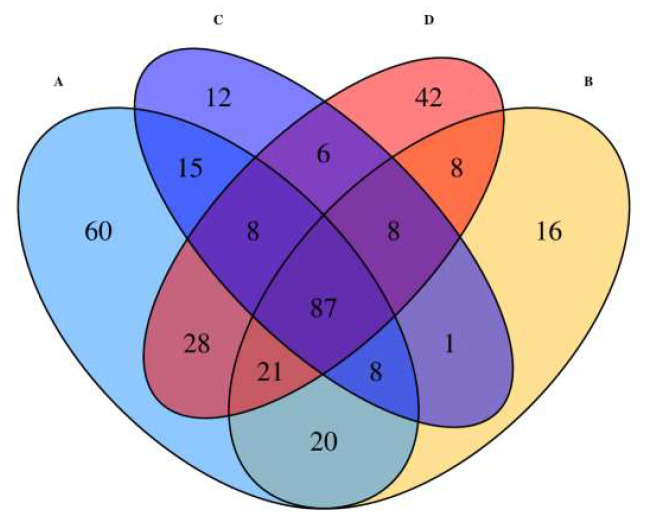
Distribution of shared and unique bacterial genera in the gut microbiota of *C. quadricarinatus* under PS-NP stress. Values indicate the number of genera specific to individual groups or shared among group combinations following exposure to PS-NPs. The control (A) and long-term exposure (D) groups harbored more unique taxa (12 and 20, respectively) compared to the mid-term exposure groups B (15) and C (21). A core of 87 genera was conserved across all groups.

Genus-level compositional analysis revealed marked inter-group variation in the distribution of unique and shared taxa. The number of unique genera was 60 in Group A (control), 16 in Group B (1-week exposure), 12 in Group C (2-week exposure), and 42 in Group D (3-week exposure), while a core microbiota comprising 87 genera was conserved across all treatment groups (Figure 4).

Prolonged PS-NP exposure elicited a pronounced decline in alpha diversity. Specifically, the ACE, Chao1, and Shannon indices were significantly elevated in Group A relative to Group D (*p* < 0.05), indicating higher species richness and evenness under control conditions. Conversely, the Simpson index, which increases as community evenness decreases, was significantly lower in Group A compared to all exposed groups and attained its maximum in Group D (*p* < 0.05). Collectively, these metrics substantiate a time-dependent erosion of both taxonomic richness and community evenness under sustained PS-NP challenge (Table 1).

**Figure 5:**
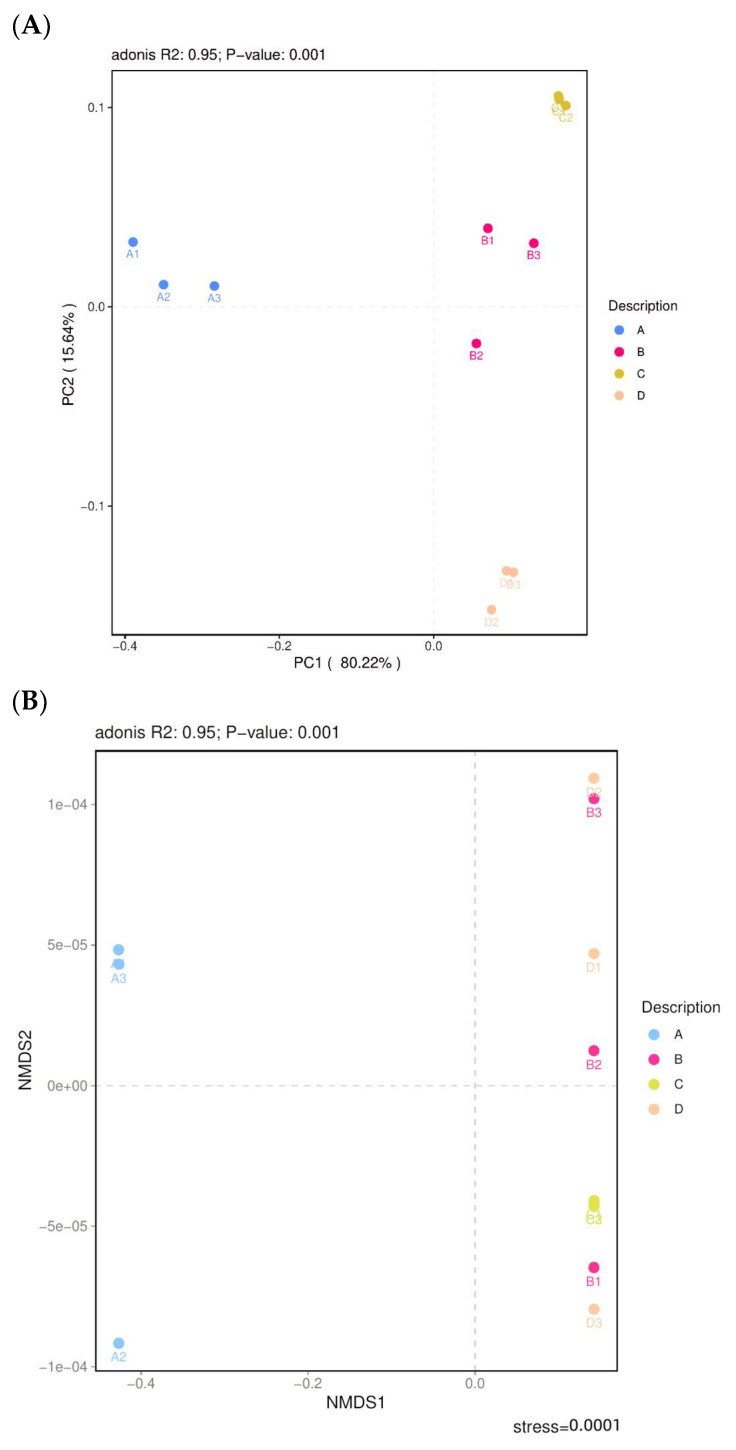
Beta-diversity analysis of gut microbiota in *C. quadricarinatus* under PS-NP stress. Community composition was visualized using (**A**) PCoA and (**B**) NMDS. Samples cluster distinctly by treatment group (A: control, B: 1-week, C: 2-week, D: 3-week exposure), demonstrating a time-dependent restructuring. PERMANOVA indicates exposure time accounts for 95% of the observed variation (*p* = 0.001).

Beta diversity, assessed via principal coordinate analysis (PCoA) based on weighted UniFrac distances and non-metric multidimensional scaling (NMDS) based on Bray–Curtis dissimilarities, revealed pronounced restructuring of the gut microbial communities. Both ordination methods yielded congruent results: samples from different exposure groups formed distinct, non-overlapping clusters, demonstrating that PS-NP exposure significantly reconfigures the overall architecture of the intestinal microbiota (Figure 5).

#### 3.4.2. Microbial Community Composition

**Figure 6:**
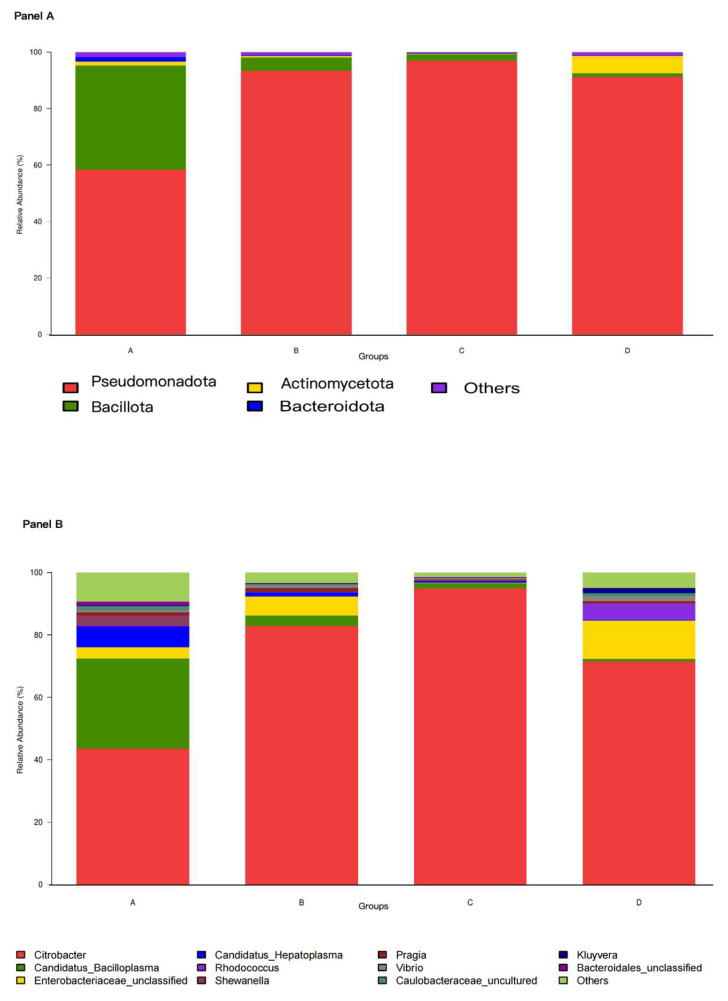
Distribution map of dominant bacterial species at the phylum level (**A**) and genus level (**B**). Note: The horizontal axis represents the sample name. The vertical axis represents the relative abundance percentage. Different colors represent different species.

PS-NP exposure induced marked compositional shifts in the gut microbiota at both phylum and genus levels (Figure 6).

At the phylum level, Pseudomonadota constituted the dominant taxon across all groups. In Groups A (control), B (1-week), and C (2-week), Bacillota ranked as the second most abundant phylum. However, by week 3 (Group D), Actinomycetota supplanted Bacillota as the secondary dominant phylum. This transition reflected a general trend of progressive Pseudomonadota enrichment and Bacillota depletion over the exposure period, although Pseudomonadota abundance exhibited a modest attenuation at the 3-week time point.

At the genus level, *Citrobacter* was highly abundant across all groups and its abundance increased with exposure duration from week 1 to 2. Notably, in Group D (3-week), the abundance of *Citrobacter* decreased. Conversely, *Candidatus* Bacilloplasma, which was prevalent in the control group, exhibited a gradual decline with increasing stress time. By week 3, the abundances of Enterobacteriaceae (family level) and *Rhodococcus* increased markedly in Group D.

**Figure 7:**
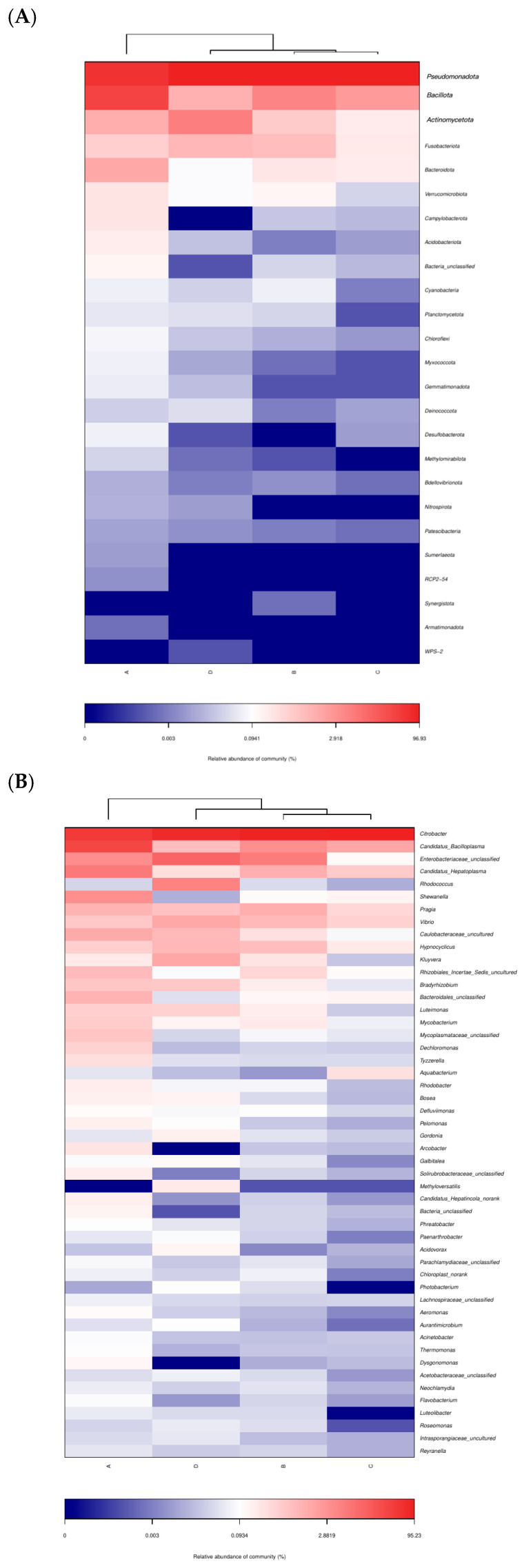
Species abundance clustering heatmap at the phylum level (**A**) and genus level (**B**). Note: Horizontal representation of samples and groups, vertical representation of species taxa. The clustering tree on the left is a species clustering tree, and the clustering tree above is a sample clustering tree From blue to red, it indicates the relative proportion of a species among different species, from low to high.

Cluster analysis of species abundance further delineated these community shifts (Figure 7). At the phylum level, Group A clustered mainly with Pseudomonadota and Bacillota, while Groups B, C, and D were primarily associated with Pseudomonadota. At the genus level, Group A clustered with *Citrobacter* and *Candidatus* Bacilloplasma; Group B with *Citrobacter* and secondarily with Enterobacteriaceae; and Groups C and D predominantly with *Citrobacter*.

In summary, PS-NPs elicited a time-dependent restructuring of the gut microbiota characterized by an initial expansion of Pseudomonadota and *Citrobacter* concomitant with the decline of Bacillota and *Candidatus* Bacilloplasma. Following three weeks of exposure, the abundances of Pseudomonadota and *Citrobacter* decreased, while Enterobacteriaceae and *Rhodococcus* emerged as the dominant taxa.

#### 3.4.3. Linear Discriminant Analysis Effect Size (LEfSe) Analysis and Functional Prediction of Gut Microbiota

To identify taxonomic biomarkers associated with PS-NP stress, LEfSe was performed. The analysis revealed distinct, time-resolved microbial signatures for each exposure group (Supplementary Figure S1).

In the control group (A), the significantly enriched taxa included *Shewanella, Caulobacteraceae, Bradyrhizobium, Xanthomonadaceae, Mycoplasmataceae, Luteimonas, Protochlamydia,* and *Arcobacter*. Following one week of exposure (Group B), only *Oceanisphaera* was significantly enriched. After two weeks (Group C), *Citrobacter* and *Aquabacterium* emerged as the characteristic genera. In the three-week exposure group (D), the enriched taxa were primarily affiliated with the class GammaPseudomonadota, including *Enterobacteriaceae, Rhodococcus, Kluyvera, Vibrio, Hypnocyclicus,* and *Methyloversatilis*. These stress-responsive genera are proposed as potential biomarkers for monitoring PS-NP exposure.

Functional profiling based on KEGG pathway annotation identified 41 level-2 functional categories. Comparative analysis revealed that the most pronounced differences among groups were concentrated within three major pathway hierarchies: Metabolism (including carbohydrate, amino acid, and energy metabolism), Environmental Information Processing (predominantly membrane transport), and Genetic Information Processing (comprising replication and repair, and translation). Among these, Metabolism and Membrane Transport (within Environmental Information Processing) represented the core functional shifts in the gut microbiota under PS-NP stress (Supplementary Figure S1).

### 3.5. Enrichment of Microplastics in the Hepatopancreas Following PS-NP Stress

Hepatopancreatic nanoplastic burdens were quantitatively assessed using Py-GC/MS. Targeted analysis revealed substantial enrichment of PS-NPs following prolonged exposure (Table 2). The PS-NP concentration in the control group (Group A) was 6.94 μg/g, which increased markedly to 65.38 μg/g after three weeks of exposure (Group D), corresponding to a 9.4-fold accumulation.

Beyond PS-NPs, additional polymer types were detected in the hepatopancreas (Table 2). Concentrations of polyethylene (PE), polyvinyl chloride (PVC), and polyamide 66 (PA66) were elevated in Group D (1.05, 3.16, and 0.53 μg/g, respectively) relative to Group A (0, 2.09, and 0 μg/g, respectively). In contrast, polypropylene (PP) exhibited a distinct temporal pattern, with a marginally lower concentration in Group D (0.04 μg/g) compared to Group A (0.13 μg/g). Collectively, these findings corroborate that the hepatopancreas serves as a primary sink for the bioaccumulation of diverse microplastic polymers, with PS-NPs displaying the most pronounced time-dependent increase.

The average accumulation rate of PS-NPs in the hepatopancreas over the 3-week exposure period was approximately 19.48 μg/g per week.

## 4. Discussion

### 4.1. Effects of PS-NPs on Hepatopancreas Antioxidant, Immune-Related Enzymes, and Histology in C. quadricarinatus

In the present study, hepatopancreatic SOD and GPx activities exhibited a biphasic temporal pattern (initial induction followed by significant attenuation), with the nadir observed in the 3-week exposure group (Group D). As SOD and GPx are integral components of crustacean non-specific immune defense and serve as sensitive sentinels of oxidative status [19,20], this response profile is consistent with a classical hormetic adaptation to xenobiotic challenge. The early elevation of these enzymatic activities likely reflects compensatory upregulation of the antioxidant machinery to mitigate reactive oxygen species (ROS) overgeneration triggered by PS-NP internalization [12,15]. Comparable hormetic responses have been documented in *L. vannamei* following microplastic exposure [12,14]. However, the subsequent decline under prolonged exposure suggests exhaustion of antioxidant capacity, potentially culminating in oxidative injury and disruption of redox homeostasis. This interpretation is supported by the significantly depressed SOD activity in Group D relative to all other treatment groups, implying functional collapse of the hepatopancreatic detoxification system under sustained PS-NP insult.

Concomitantly, AKP activity, a key enzymatic marker of innate immunity and phosphate metabolism [21], exhibited a progressive, time-dependent decrease and reached its minimum in Group D. In contrast, ACP activity remained unaltered across groups. This differential responsiveness likely reflects distinct physiological roles and differential susceptibilities of these two hydrolases to toxicant exposure. The sustained suppression of AKP activity denotes time-cumulative impairment of non-specific immune function within the hepatopancreas. AKP is predominantly localized in hepatopancreatic tissues, and its activity fluctuation serves as a reliable indicator of immune status. Previous investigations have demonstrated that under stress conditions, AKP activity in *L. vannamei* hepatopancreas undergoes time-dependent modulation, reflecting dynamic metabolic reallocation and energy partitioning [21,22]; its downregulation has been mechanistically linked to hepatocellular injury and immunotoxicity in decapod crustaceans [15]. The pronounced AKP inhibition observed herein coincides temporally with substantial PS-NP accumulation (65.38 μg/g) and concomitant enrichment of other polymer types in Group D hepatopancreas, consistent with a possible causal relationship between particle burden and enzymatic dysfunction.

Histopathological examination further corroborated the biochemical findings [23]. In the control group (Group A), hepatic tubules exhibited intact basement membranes, clearly defined boundaries, and a star-shaped central lumen, with normally structured and tightly arranged hepatopancreatic cells. After one week of exposure (Group B), B cells (secretory cells) showed increased volume, but overall tubule architecture remained relatively normal. By week 2 (Group C), hepatic tubules became significantly enlarged and flattened, with increased numbers and sizes of intracellular vacuoles. Cell arrangement became disordered, and B cells exhibited marked increases in volume and in the number of transport vesicles, some containing visible particulate matter. After three weeks (Group D), the damage progressed further: tubule boundaries became blurred, cellular arrangement was severely disrupted, hepatopancreatic cells showed signs of lysis, and cell numbers were markedly reduced. This progressive histopathological deterioration parallels the time-dependent decline in antioxidant and immune enzyme activities, providing direct morphological evidence of PS-NP-induced hepatopancreatic injury [24,25,26].

Furthermore, the observed enzymatic and histological perturbations may be indirectly potentiated by PS-MP-induced gut dysbiosis. Our microbiota profiling revealed significant depletion of beneficial Bacillota and concurrent expansion of opportunistic pathobionts (Enterobacteriaceae, *Rhodococcus*) in Group D. Disruption of intestinal barrier integrity and alteration of microbial metabolite profiles can trigger systemic inflammatory cascades and compromise hepatopancreatic function via the gut–hepatopancreas axis, a recognized physiological conduit in aquatic invertebrates [12]. Accordingly, the suppression of AKP activity and the observed tissue damage likely emanate from a synergistic interplay between direct particle cytotoxicity and indirect effects mediated through intestinal microbial imbalance.

Collectively, the time-dependent modulation of antioxidant enzymes (SOD, GPx), the sustained inhibition of AKP activity, and the progressive histopathological lesions demonstrate that chronic exposure to 100 mg/L PS-NPs elicits oxidative stress, immunosuppression, and structural damage in the hepatopancreas of *C. quadricarinatus*. These findings underscore the vulnerability of this pivotal metabolic organ to nanoplastic contamination and highlight the need for mechanistic studies elucidating the molecular pathways linking oxidative damage, immune dysfunction, and tissue pathology.

### 4.2. Effects of PS-NPs on the Gut Microbiota of C. quadricarinatus

PS-NP exposure induced pronounced time-dependent dysbiosis in the gut microbiota of *C. quadricarinatus*, characterized by progressive depletion of beneficial taxa, enrichment of opportunistic pathobionts, and marked attenuation of alpha diversity. These observations reinforce the concept that the intestinal microbiome constitutes a sensitive and integrative target of microplastic-induced toxicity.

#### 4.2.1. Shifts in Gut Microbial Composition and Ecological Implications

The sustained decline in Bacillota, key contributors to energy homeostasis and short-chain fatty acid production [27,28], likely reflects compromised intestinal energy acquisition. Conversely, Pseudomonadota (particularly Enterobacteriaceae and *Citrobacter*) are typical dysbiotic signatures of environmental stress [29,30]. The transient proliferation of *Citrobacter* during intermediate exposure (Groups B and C), followed by its decline and the concurrent emergence of Enterobacteriaceae and *Rhodococcus* at week 3, delineates a successional trajectory of opportunistic taxa, indicating progressive erosion of colonization resistance.

The marked proliferation of *Rhodococcus* in Group D warrants attention. While *Rhodococcus* species are renowned for degrading xenobiotics (e.g., polyethylene via laccase) [31,32], their bloom at the expense of core commensals (e.g., Bacillota) raises concerns as opportunistic pathobionts. The simultaneous enrichment of Enterobacteriaceae and *Vibrio* in Group D further substantiates the concept that late-stage dysbiosis is characterized by expansion of multiple potentially pathogenic taxa [29,33]. Therefore, while *Rhodococcus* holds promise for ex situ bioremediation applications, its in situ bloom within the crustacean gut should be interpreted as a marker of severe microbial disequilibrium rather than a beneficial event.

*Candidatus* Bacilloplasma, a commensal associated with the hindgut epithelium of terrestrial isopods and aquatic crustaceans [34], progressively declined under PS-NP stress, consistent with reports in *P. clarkii* exposed to nanoplastics [16]. Its loss may serve as a sensitive indicator of gut health deterioration in crustaceans [28].

#### 4.2.2. Loss of Diversity and Identification of Time-Specific Microbial Biomarkers

Alpha diversity metrics (ACE, Chao1, Shannon) were significantly reduced in Group D relative to controls, whereas the Simpson index was elevated, collectively indicating erosion of both species richness and community evenness. This pattern aligns with findings by Yaripour et al., who reported diminished bacterial diversity in the freshwater amphipod Gammarus pulex following high-concentration nanoplastic exposure [35]. Such simplification compromises functional redundancy and host resistance to pathogens [36]. The partial recovery of unique genera counts in Group D observed in our core microbiota analysis should not be misinterpreted as restoration; rather, it likely reflects emergence of a distinct, low-diversity community dominated by stress-adapted taxa.

LEfSe analysis identified distinct time-specific biomarkers [37,38]. In controls (Group A), enriched taxa comprised a functionally diverse assemblage including *Shewanella, Caulobacteraceae, Bradyrhizobium, Xanthomonadaceae, Mycoplasmataceae, Luteimonas, Protochlamydia,* and *Arcobacter*, indicative of a stable commensal consortium [39,40]. Following one week of exposure (Group B), exclusive enrichment of *Oceanisphaera* suggested an acute, selective filtering effect. After two weeks (Group C), *Citrobacter* and *Aquabacterium* emerged as characteristic genera; both have been associated with dysbiosis and altered redox conditions in stressed aquatic organisms [30,41]. Following three-week exposure (Group D), the biomarker profile shifted dramatically toward GammaProteobacteria-affiliated taxa, encompassing Enterobacteriaceae, *Rhodococcus, Kluyvera, Vibrio, Hypnocyclicus,* and *Methyloversatilis*. This constellation of pathobionts indicates collapse of colonization resistance and severe late-stage dysbiosis [16,29,33]. KEGG functional prediction revealed shifts in Metabolism (carbohydrate, amino acid, and energy metabolism), Environmental Information Processing (predominantly membrane transport), and Genetic Information Processing (replication and repair, translation) [42,43]. Among these, membrane transport pathways, particularly ABC transporters and secretion systems, emerged as core functional shifts, likely reflecting microbial adaptation to xenobiotic stress and nutrient scarcity within the compromised gut environment [44,45].

#### 4.2.3. Linking Gut Dysbiosis to Hepatopancreatic Toxicity: The Gut–Hepatopancreas Axis

Temporal coincidence of severe dysbiosis, maximal PS-NP accumulation (65.38 μg/g), AKP suppression, and histopathological lesions in Group D is consistent with a gut–hepatopancreas axis [40]. Disruption of intestinal barrier integrity, potentially exacerbated by loss of epithelial-associated *Candidatus* Bacilloplasma and expansion of pro-inflammatory Enterobacteriaceae, may facilitate translocation of particles or endotoxins, thereby imposing additional detoxification burden on the hepatopancreas. Investigations in *E. sinensis* have demonstrated that nanoplastic exposure induces time-dependent hepatopancreatic accumulation and activates antioxidant and immune-related pathways [46,47]. Similar findings have been reported in *L. vannamei* [12,14], supporting the operation of a gut–hepatopancreas axis in crustaceans.

In summary, chronic exposure to 100 mg/L PS-NPs induced time-dependent restructuring of the gut microbiota in *C. quadricarinatus*, characterized by depletion of beneficial commensals (Bacillota, *Candidatus* Bacilloplasma), transient *Citrobacter* blooms, and eventual dominance of Enterobacteriaceae and *Rhodococcus*. Concomitant loss of alpha diversity and emergence of a low-richness, pathogen-enriched community signify severe dysbiosis. These changes were coupled with significant PS-NP accumulation, immunosuppression, and progressive histopathological damage in the hepatopancreas, consistent with the operation of a gut–hepatopancreas axis. We acknowledge that all crayfish were fasted during exposure to eliminate dietary interference. Starvation alone can affect gut microbiota and tissue metabolism; however, because the control group was fasted identically, the comparative differences remain valid indicators of PS-NP toxicity. Future studies should incorporate pair-feeding or controlled feeding protocols.

### 4.3. Enrichment of PS-NPs and Other Microplastics in the Hepatopancreas

Pyrolysis gas chromatography–mass spectrometry (Py-GC/MS) was employed for quantitative determination of PS-NPs and additional polymer types in *C. quadricarinatus* hepatopancreas. This analytical platform enables robust identification and quantification of microplastics based on characteristic thermal degradation products, offering high specificity and sensitivity for complex biological matrices [48].

Following three-week exposure to 100 mg/L PS-NPs, hepatopancreatic PS-NP content reached 65.38 μg/g, representing a 9.4-fold increase over control levels (6.94 μg/g). This pronounced accumulation demonstrates a clear time-dependent bioaccumulation trajectory. Concurrently, elevated concentrations of other microplastics (including PE, PVC, and PA66) were detected in Group D, indicating that the hepatopancreas functions as a non-selective sink for diverse plastic debris. These observations align with previous reports documenting preferential microplastic accumulation in crustacean hepatopancreas attributable to its central roles in digestion, detoxification, and lipid metabolism [2,49].

The hepatopancreas, as the largest metabolic organ in decapod crustaceans, exhibits heightened susceptibility to particle retention. Its tubular architecture and endocytic capacity facilitate internalization of nano- and micro- scale particles, while sluggish turnover of stored lipids may prolong particle residence time [15]. Moreover, PS-NPs have been demonstrated to induce oxidative stress and lysosomal membrane destabilization in hepatopancreatic cells, potentially impairing particle clearance and exacerbating accumulation [17]. Thus, the substantial enrichment observed in Group D likely reflects both sustained uptake and diminished elimination capacity under chronic stress. This interpretation is further supported by the histopathological evidence of cellular lysis and disorganization, which may compromise the organ’s ability to eliminate internalized particles.

As benthic opportunistic feeders, redclaw crayfish are particularly prone to ingestion of sediment-associated microplastics [2]. Given the environmental persistence and continuous fragmentation of larger plastic debris, PS-NP contamination in aquatic systems is projected to escalate for decades [3]. This underscores the relevance of *C. quadricarinatus* as both a sentinel species for plastic pollution monitoring and a potential vector for trophic transfer.

The bioaccumulation of microplastics in edible tissues raises legitimate food safety concerns. Human exposure to microplastics occurs primarily through dietary intake, and consumption of contaminated seafood, particularly visceral organs such as the hepatopancreas, may pose non-negligible health risks [18]. Microplastics can elicit physical injuries (e.g., intestinal obstruction, epithelial abrasion) and chemical toxicity via leaching of plastic additives or adsorbed environmental pollutants, including endocrine-disrupting chemicals [3]. Emerging evidence also suggests that ingested microplastics can accumulate in human organs (lungs, liver, intestine, brain) and may be implicated in chronic inflammation and carcinogenesis [18]. Although direct causal evidence in humans remains limited, the precautionary principle warrants judicious consideration.

Several limitations should be acknowledged. Microplastic quantification was performed only for the control and 3-week exposure groups; the absence of data from weeks 1 and 2 (Groups B and C) limits the temporal resolution of the bioaccumulation trajectory. Nevertheless, the significant 9.4-fold increase observed by week 3 clearly demonstrates substantial net accumulation. Future studies should include all time points to establish a complete uptake and depuration time-course. The presence of PS-NPs in control crayfish (6.94 μg/g) also deserves comment. Possible sources include: (1) residual particles in laboratory water despite pre-filtration (0.45 μm); (2) airborne deposition during handling or processing; (3) trace contamination from plastic laboratory consumables (e.g., tubing, tanks); or (4) pre-experimental exposure at the commercial breeding facility, where microplastics are not routinely monitored. Analytical detection limits (0.1 μg/g) and potential carry-over during Py-GC/MS cannot be excluded. Critically, the control level served as a consistent baseline, and the 9.4-fold higher concentration in Group D far exceeds any plausible background fluctuation, demonstrating true net accumulation attributable to PS-NP exposure. Moreover, identical analytical and procedural blanks (consistently below detection) were applied to both control and exposed samples, ensuring that the reported difference is not an artifact. Hence, background contamination does not undermine the conclusion that chronic PS-NP exposure leads to substantial additional bioaccumulation.

Only a single concentration (100 mg/L) was tested, selected for hazard identification and mechanistic exploration. This level is higher than typical environmental concentrations; therefore, the results should be interpreted as evidence of potential toxicity rather than a direct quantitative risk assessment for real-world scenarios. Dose–response studies with multiple lower concentrations are needed to determine no-observed-effect levels and to better inform ecological risk assessment. The calculated average accumulation rate (19.48 μg/g per week) is a linear approximation; because intermediate time points were not analysed, true accumulation kinetics could be non-linear (e.g., faster initial uptake followed by slower net gain). Full time-course sampling is required to characterise precise accumulation dynamics.

Based on the substantial PS-NP enrichment documented herein, we recommend consumer vigilance regarding consumption of *C. quadricarinatus* hepatopancreas sourced from potentially contaminated waters. Future research should prioritize elucidation of trophic transfer dynamics in freshwater food webs, development of rapid monitoring methodologies for microplastic contamination in aquaculture products, and comprehensive assessment of long-term health implications associated with chronic dietary exposure to environmentally relevant concentrations.

## 5. Conclusions

This study shows that exposure to 100 mg/L PS-NPs over three weeks induces time-dependent multi-system toxicity in *C. quadricarinatus.* It causes oxidative stress overload, progressive immunosuppression, and histopathological damage (tubule enlargement, vacuolation, B cell hypertrophy, and cell lysis) in the hepatopancreas. The gut microbiota undergoes severe dysbiosis, characterized by depletion of beneficial taxa (Bacillota, *Candidatus* Bacilloplasma), enrichment of opportunistic pathobionts (Enterobacteriaceae, *Rhodococcus*), and reduced alpha diversity. LEfSe analysis further resolved time-specific bacterial biomarkers (*Citrobacter* at week 2; Enterobacteriaceae and *Rhodococcus* at week 3), offering sensitive indicators for tracking dysbiosis progression. The hepatopancreas accumulates substantial PS-NPs (9.4-fold increase, reaching 65.38 μg/g), and this peak accumulation coincided with maximal immunosuppression and gut dysbiosis, supporting a gut–hepatopancreas axis in crustaceans. These findings highlight ecological risks of nanoplastic contamination. Given that the hepatopancreas is a frequently consumed visceral organ, heightened awareness regarding human dietary exposure to crayfish harvested from PS-NP-polluted environments is warranted.

## Figures and Tables

**Table 1:** Estimation of gut microbiota richness (Chao1 and ACE) and diversity (Shannon and Simpson indices) for each group.

	Group A	Group B	Group C	Group D
Richness estimate				
ACE	271.25 ± 22.91 ^a^	248.25 ± 22.34 ^ab^	201.50 ± 29.55 ^ab^	187.50 ± 20.42 ^b^
Chao1	276.75 ± 28.34 ^a^	239.25 ± 20.07 ^ab^	223.75 ± 30.63 ^ab^	185.50 ± 23.12 ^b^
Diversity estimators				
Shannon	2.33 ± 0.10 ^a^	1.61 ± 0.05 ^ab^	1.41 ± 0.06 ^ab^	0.79 ± 0.02 ^b^
Simps on	0.20 ± 0.01 ^a^	0.36 ± 0.01 ^b^	0.44 ± 0.03 ^b^	0.64 ± 0.01 ^c^

ACE is an abundance-based coverage estimation, with data presented as mean ± standard deviation. Different superscripts in the same row indicate significant differences (*p* < 0.05).

**Table 2:** Concentrations of polystyrene and other microplastics (PE, PP, PVC, PA66) in the hepatopancreas of *C. quadricarinatus* from Group A (control) and Group D (3-week exposure).

Group	PS MP/μg/g	PE/μg/g	PP/μg/g	PVC/μg/g	PA66/μg/g
A	6.94	0	0.13	2.09	0
D	65.38	1.05	0.04	3.16	0.53

## Data Availability

The data and materials are available on request.

## Supplementary Materials

The following supporting information can be downloaded at: https://www.mdpi.com/article/10.3390/ani16131977/s1, Figure S1. Microbial biomarkers and predicted functional shifts in the gut microbiota of *C. quadricarinatus* under PS-NPs stress. Panel A and B: Cladogram from LEfSe analysis. The diagram shows the phylogenetic distribution of bacterial taxa significantly enriched in each treatment group. Colored nodes represent taxa enriched in the corresponding group (red: control/Group A; green: 1-week exposure/Group B; blue: 2-week exposure/Group C; purple: 3-week exposure/Group D). Panel C: Predicted functional profiles based on KEGG pathway analysis (Level 2). The heatmap displays the relative abundance of key functional categories across groups. Major differentially abundant pathways include those involved in Metabolism (e.g., Carbohydrate and Amino Acid Metabolism), Environmental Information Processing (predominantly Membrane Transport), and Genetic Information Processing.
