# Supplementary material for: Time-Dependent Polystyrene Nanoplastic Toxicity in Cherax quadricarinatus: Oxidative Stress, Gut Dysbiosis, and Hepatopancreatic Bioaccumulation
Source: Animals (Basel). 2026 Jun 26;16(13):1977. doi: 10.3390/ani16131977 (PMC13360381; doi:10.3390/ani16131977)
Supplement: Supplementary file 1 [file animals-16-01977-s001.zip › animals-4376252-supplementary.pdf]

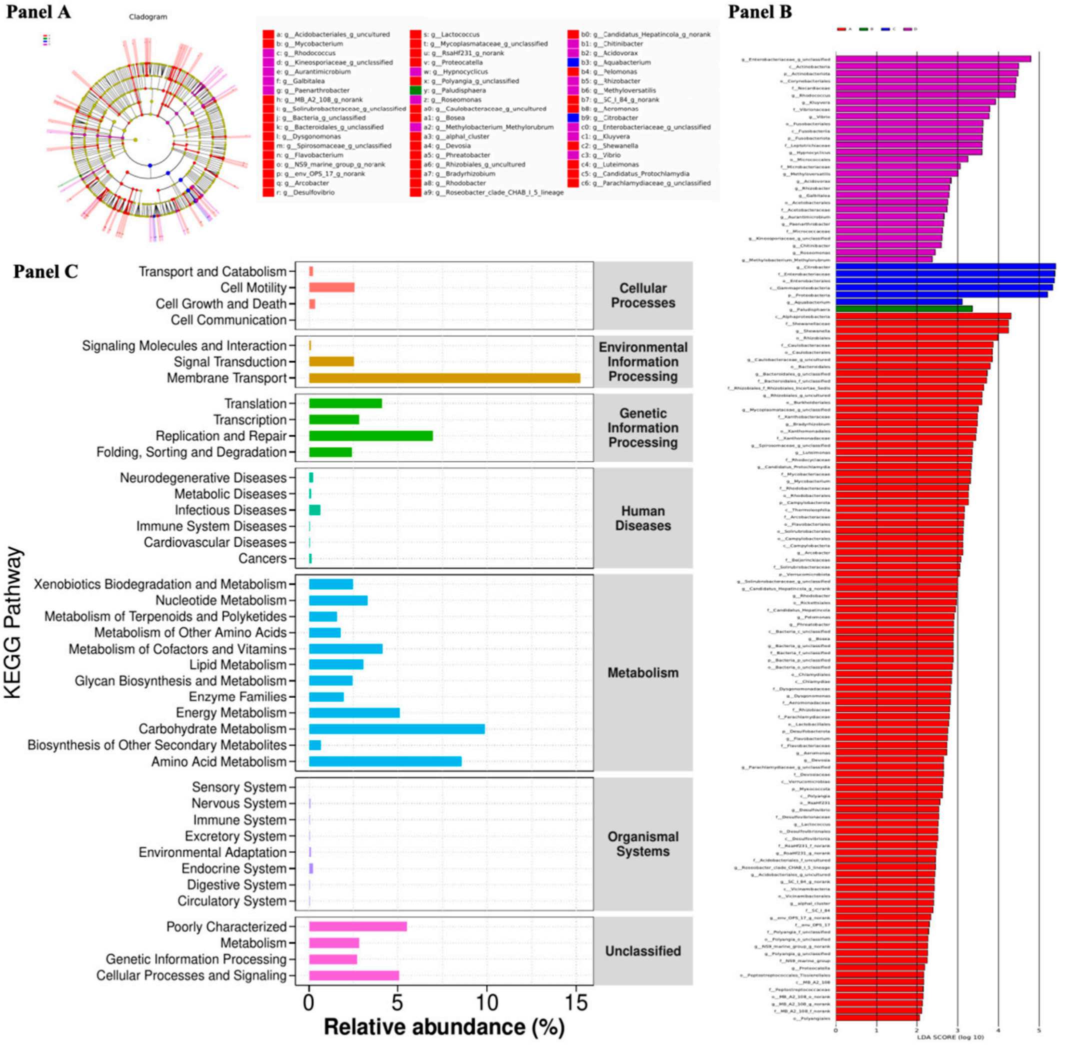

Supplementary Fig. S1. Microbial biomarkers and predicted functional shifts in the gut microbiota of *C. quadricarinatus* under PS-NPs stress. Panel A and B: Cladogram from LEfSe analysis. The diagram shows the phylogenetic distribution of bacterial taxa significantly enriched in each treatment group. Colored nodes represent taxa enriched in the corresponding group (red: control/Group A; green: 1-week exposure/Group B; blue: 2-week exposure/Group C; purple: 3-week exposure/Group D). Panel C: Predicted functional profiles based on KEGG pathway analysis (Level 2). The heatmap displays the relative abundance of key functional categories across groups. Major differentially abundant pathways include those involved in Metabolism (e.g., Carbohydrate and Amino Acid Metabolism), Environmental Information Processing (predominantly Membrane Transport), and Genetic Information Processing.
